# System, institutional, and client-level factors associated with formal healthcare utilisation among older adults with low income under a social protection scheme in Ghana

**DOI:** 10.1186/s13690-023-01063-w

**Published:** 2023-04-24

**Authors:** Williams Agyemang-Duah, Dennis Asante, Joseph Oduro Appiah, Anthony Kwame Morgan, Isaac Verberk Mensah, Prince Peprah, Anthony Acquah Mensah

**Affiliations:** 1 Department of Geography and Planning, Queen’s University, Kingston, Canada; 2 College of Medicine & Public Health, Rural and Remote Health, Flinders University, Adelaide, South Australia Australia; 3 Department of Geography, Environment & Spatial Analysis, Cal Poly Humboldt, Arcata, California, United States; 4 Department of Planning, Kwame Nkrumah University of Science and Technology, Kumasi, Ghana; 5 Department of Social Sciences, St. Ambrose College of Education, Wamfie, Bono Region Ghana; 6 Social Policy Research Centre, Centre for Primary Health Care and Equity, University of New South Wales, Sydney, Australia

**Keywords:** System factors, Institutional factors, Client preference factors, Formal healthcare utilisation, Older adults with low income, Social protection scheme, Ghana

## Abstract

**Background:**

In sub-Saharan African context, effect of system, institutional and client-level factors on formal healthcare utilisation among older adults with low income, especially those under a social protection scheme (called Livelihood Empowerment against Poverty [LEAP] programme) is least explored in the literature. However, an adequate understanding of how these factors contribute to formal healthcare utilisation among older adults who are classified as poor (in terms of low income) is important to inform health policy decisions. The aim of this study, therefore, was to examine the contributions of system, institutional and client-level factors in formal healthcare utilisation among older adults with low income under the LEAP programme in Ghana.

**Methods:**

Data associated with this study were obtained from an Ageing, Health, Lifestyle and Health Services survey conducted between 1 and 20 June 2018 (*N* = 200) in the Atwima Nwabiagya Municipal and Atwima Nwabiagya North District of Ghana. Multivariable logistic regressions were used to determine system, institutional and client-level factors associated with formal healthcare utilisation among older adults with low income under the LEAP programme in Ghana. The significance of the test was set at a probability value of 0.05 or below.

**Results:**

The study revealed that participants who relied on the LEAP programme and/or health insurance subscription to cater for their healthcare expenses (AOR: 11.934, CI: 1.151-123.777), those whose family/caregivers decided on when and where to use formal healthcare (AOR:12.409; CI: 2.198–70.076) and those who did not encounter communication problem with healthcare providers (AOR: 1.358; CI: 1.074–3.737) were significantly more likely to utilise formal healthcare services compared with their counterparts. The study further found that participants who perceived the attitude of healthcare providers as poor (AOR: 0.889; CI: 0.24–0.931) and those who spent 20–40 minutes at the healthcare facility were significantly less likely to utilise formal healthcare services compared with their counterparts (AOR: 0.070; CI: 0.006–0.195).

**Conclusion:**

Our findings suggest that reducing waiting time at healthcare facilities, improving social protection and/or health insurance schemes, improving patient-doctor communication and promoting attitudinal change programmes (such as orientations and supportive supervision) for healthcare providers may help to facilitate the use of needed formal healthcare services by older adults with low income in Ghana.

## Background

The population of older adults is increasing at a pace that continues to engage policy attention worldwide. Currently, older people aged 60 years or above are estimated to be around 900 million across the globe and this subpopulation is expected to outnumber children for the first time in world history by 2047 [1]. Based on the current growth rates of older individuals, the geographical distribution of the ageing demographics is projected to reach 9% in Africa, 24% in Asia, and 25% in Latin America. The proportion of older adults in North America and Europe is expected to be 28% and 35%, respectively by 2050 [2]. In sub-Saharan Africa [SSA], Ghana is identified as one of the countries with the fastest ageing population. The proportion of the country’s older demographic (60+) is estimated to rise from 6.9% in 2015 to 12% by 2050 [3, 4]. The expanding segments of the older population have been attributed to factors such as declining fertility and mortality rates and advanced technology and biomedical breakthroughs that have led to increased life expectancy/longevity [5].

Ageing has been associated with an increased risk of chronic conditions and functional disabilities. Frailty, mobility challenges, multimorbidity, and mental health challenges are reported to be more prevalent in older adult population [6, 7]. Even though ageing is not synonymous with poor health or chronic conditions, it is associated with health conditions such as diabetes, cardiovascular diseases, kidney problems due to physiological changes and gradual biological decline along the life course [7]. Thus, population ageing and its associated higher risk of chronic morbidities place older adults on a greater need for health services to improve health outcomes and general well-being in later life [6, 8, 9]. Hence, older adults are higher users of health services compared to the younger age groups [10]. For example, while about 52% of the residents in Ghana utilise various healthcare services every year, older adults constitute about 52.41% of the annual health service users in the country [3, 11]. This presupposes that population ageing may exert additional pressure on healthcare resources as well as increase the cost burden of health service provision. In examining the relationship between ageing, health services use, and healthcare expenditure, Breyer and colleagues [12] reported a positive correlation between ageing and healthcare expenditure. A study by Ambagtsheer and Moussa [13] in Côte d’Ivoire revealed that frailty was associated with increased health service use and total health expenditure. Awoke et al. [11] indicated that ageing has a positive relationship with the use of formal healthcare services in Ghana. Similarly, Huang et al. [14] noted that major depressive symptoms predominantly common in older adults increased the healthcare utilization and cost in Sao Paulo. Older adults with comorbid physical and mental health conditions were more likely to be hospitalized or receive emergency services in the US [15]. Despite their higher health services demand, not all older adults can access required health services at the time of need, an idea suggesting determinants of health services use and outcomes [16].

Considerable literature has emerged describing demographic, biological, and socioeconomic factors that contribute to health services uptake among older adults [11, 17]. Older adults with better economic conditions tend to access and utilize health services easily and more desirably [17, 18]. This evidence suggests existential barriers to access and use of health services needed by older people with lower socioeconomic status. The inequality of access and use of desired health services, according to Van Doorslaer et al. [19, 20, 21] exacerbates for poor individuals in healthcare systems where users pay out-of-pocket for healthcare services and/or episode billing systems (payments at the point of accessing services) [20, 22]. Thus, the affordability of healthcare becomes prohibitive for lower socioeconomic groups, thereby preventing them from utilizing needed health services [23]. Socio-demographic factors such as age, gender, education, and marital status have been verified in several studies to predict health services use among older populations [24, 25]. In China, Gong et al. [10] examined the impact of marital status, gender, and age on healthcare resource use. The study observed a significant association between the study variables and healthcare utilisation. They specifically highlighted that married older females had higher odds than their male counterparts to access available health services. Moreover, another potential determinant of healthcare access by older adults is discrimination perpetuated by clinical staff or administrative and clerical staff. Discrimination can manifest in a form of unequal treatment in healthcare settings by virtue of an individual’s membership in a socially defined group [26, 27]. Communication gaps between providers and patients as well as the differential approach in physician referral behaviour may help in explaining the differences in patterns of health services uptake by older people [28].

Despite the expanding literature on determinants of health services utilisation among older individuals, studies on the subject in most developing countries such as Ghana have overly focused on demographic, economic and health-related factors, neglecting point of access and systemic factors that in concert, shape the help-seeking decisions by older people. To holistically understand healthcare use determinants and to strategically design policy frameworks to achieve healthy ageing, analysis of all the possible determining factors of health services use is desirable. Therefore, this study investigates the system, institutional and client-level factors associated with formal healthcare utilisation among older adults with low income in Ghana.

## Methods

### Sampling procedure and data collection

Data associated with this study were obtained from a larger Ageing, Health, Lifestyle and Health Services (AHLHS) survey on determinants of healthcare utilisation among older adults with low income. The AHLHS survey was conducted between 01 and 20 June 2018 in the Atwima Nwabiagya District (henceforth, Atwima Nwabiagya Municipality and Atwima Nwabiagya North District) of Ghana. As a cross-sectional survey, the AHLHS survey recruited sixteen communities with distinct participants of diverse demographic and socio-economic characteristics in the Atwima Nwabiagya Municipality and Atwima Nwabiagya North District of Ghana. The survey focused on older adults 65 years or over who were enrolled in the LEAP programme. The focus on those who were 65 years or over was because of the criteria for classifying an individual as an older adult under the LEAP programme in Ghana. We highlight that since the LEAP programme is a poverty reduction programme, anyone who is enrolled in the programme is classified as poor (in terms of low income).

The survey involved 200 study participants in 16 randomly selected communities in the study district through cluster and simple random sampling techniques. The sampling and selection procedure was divided into several stages. First, a list of all LEAP programme beneficiaries was obtained from the Department of Social Welfare in the study area. Second, a total of 401 poor older people aged 65 and over were identified. Third, the required sample size (N = 200) was calculated using Miller and Brewer’s [29] sample size estimation formula. Fourth, the study district was clustered into three geographical areas; North (Abira, Boahenkwaa, Adagya, and Wurapong), Central (Kontomire, Hiawu-Besease, Kyereyease, and Apuyem), and South (Amanchia, Koben, Seidi, Fankamawe, Nkorang, and Nkaakom). Blindfolded sampling approach was used to select the required sample size allotted for each study community without replacement until the assigned sample for each community was obtained.

Interviewer-administered questionnaire was the main data collection instrument. The questionnaire for the survey was written in English however the interviews were conducted in Twi (the predominant dialect in the study communities) due to the low educational level of the participants. Responses were written in English and three graduate students from the Kwame Nkrumah University of Science and Technology, Ghana were recruited and trained to help with data collection.

### Measures

#### Outcome variable

In this study, formal healthcare utilisation was our outcome variable. Formal healthcare was defined as accessing treatment from medical practitioners at a healthcare facility (private or/and public) [30]. Formal healthcare utilisation was measured as a dichotomous variable indicating “utilisation of formal healthcare = 1” and “no utilisation of formal healthcare = 0” in the past 12 months preceding the study. This one-year period for measuring formal healthcare utilisation is consistent with previous studies [31–33].

#### Independent variables

In this study, the independent variables were grouped into system, institutional and client-level factors. The system-level factors are waiting time at the healthcare facility (1 = less than 20 min, 2 = 20–40 min, 3 = more than 40 min) [34] and healthcare costs (1 = less than GH¢ 50, 2 = GH¢50–100, 3 = More than GH¢100) [1 USD = GH¢4.76820 as at the time of the survey]. The client-level factors are sources of funds for healthcare (1 = personal, 2 = informal social networks, 3 = social protection scheme), mode of accessing healthcare (1 = walking, 2 = tricycle, 3 = vehicle), sources of healthcare information (1 = healthcare providers, 2 = informal social networks, 3 = media), a decision on when and where to use healthcare (1 = personal, 0 = family/caregiver), distance travelled to access healthcare (1 = less than 3 km, 2 = 3 or more km), type of healthcare providers consulted (1 = public health facility 2 = private health facility). The institutional factors are communication problems with healthcare providers (1 = yes, 0 = no), perception of healthcare treatment (1 = good, 2 = poor), quality of healthcare services (1 = good, 2 = poor), the attitude of healthcare providers (1 = good, 2 = poor) and satisfaction with services provided by providers (1 = good, 2 = poor). All the independent variables were measured as categorical.

### Analytical framework

In this study, both descriptive and inferential analytical techniques embedded in SPSS software version 21 (IBM, Corp, Armonk, NY, USA) were employed. Percentages and frequencies were employed to describe the demographic and socio-economic characteristics of the participants. The presence of multicollinearity was checked for independent variables using the Variance Inflation Factor (VIF), and the VIF value was less than 2.5 for all the independent variables demonstrating no strong multicollinearlity. Inferential statistics such as multivariable logistic regressions were used to estimate the association between system, institutional and client-level factors associated with formal healthcare use. We developed three separate models to explain factors associated with formal healthcare utilisation. The first model included only clients-level factors, the second model covered institutional variables plus all variables in Model 1 and the final Model (Model 3) considered system factors plus all variables in Model 2. We employed the Hosmer and Lemeshow test of homogeneity and Omnibus Tests of Model Coefficients to measure the robustness of the Models. The outcome (*P* > 0.05) of the Hosmer and Lemeshow test of homogeneity shows that the Models are a good fit for the data. The Omnibus Tests of Model Coefficients show a significant difference between the based model (without explanatory variables) and the current model with explanatory variables (*P* < 0.05). The significance of the test was set at a *p-*value of 0.05 or less.

### Ethics approval and consent to participate

Ethics clearance for this study was granted by the Committee on Human Research Publication and Ethics (CHRPE), School of Medical Sciences, Kwame Nkrumah University of Science and Technology and Komfo Anokye Teaching Hospital, Kumasi, Ghana (Ref: CHRPE/AP/311/18). Also, the purpose of the study was explained to the study participants before their informed written and verbal consents were obtained. Again, they were assured of the strict confidentiality and anonymity of the data they provided. They were further assured that their participation in the study was voluntary and that they were free to opt out at any time.

## Results

### Sample characteristics of the respondents

Table 1 presents the demographic and socio-economic characteristics of the participants. About 78% of the respondents were females, 49% were aged between 65 and 74 years, 84% were Akans, 82.5% were Christians and 26% were married. Also 62.5% had no formal education and 38% of the respondents earned GH¢100 or below a month (see Table 1).

**Table 1:** Sample characteristics of older adults with low income under a social protection scheme in Ghana, 2018

Variables Category		Count	%
Gender	Male	44	22.0
	Female	156	78.0
Age (years)	65–74	98	49.0
	75–84	52	26.0
	85 or above	50	25.0
Ethnic Group	Akan	168	84.0
	Non-Akan	32	16.0
Religion	Christian	165	82.5
	Non-Christian	35	17.5
Marital Status	Single	148	74.0
	Married	52	26.0
Education	No formal education	125	62.5
	Basic school education	54	27.0
	High school education	21	10.5
Monthly Income (GH¢)	100 or below	76	38.0
	101–200	75	37.5
	Above 200	49	24.5

**Table 2:** Descriptive analysis on client, institutional and system-level variables of formal healthcare utilization among older adults with low income under a social protection scheme in Ghana, 2018

Variables		Count (N = 200)	%
Use of formal health care for health problems in the last year	Yes*	170	85.0
	No	30	15.0
**Client factors**			
Type of health care provider	Public health facility	131	77.1
	Private health facility	39	22.9
Deciding on when and where to use healthcare	Personal	93	54.7
	Family/caregivers	77	45.3
Source of healthcare information	Healthcare provider	24	14.1
	Informal social networks	128	75.3
	Media	18	10.6
Distance to healthcare facilities	Less than 3 km	78	45.9
	3 or more km	92	54.1
Mode of accessing healthcare facility	Walking	10	5.9
	Tricycle	19	11.2
	Car/Vehicle	141	82.9
Sources of funds for healthcare	Personal	77	45.3
	informal social networks	46	27.1
	social/health protection	47	27.6
**System factors**			
Waiting time at the healthcare facility	Less than 20 min	23	13.5
	20–40 min	104	61.2
	More than 40 min	43	25.3
Healthcare costs	Less than GH ¢50	43	25.3
	GH¢ 50- GH¢100	90	52.9
	More than GH¢100	37	21.8
**Institutional factors**			
Level of treatment	Good	151	88.8
	Poor	19	11.2
Quality of care	Good	154	90.6
	Poor	16	9.4
Attitude of healthcare providers	Good	156	91.8
	Poor	14	8.2
Communication problems	Yes	99	58.2
	No	71	41.8
Healthcare use satisfaction	Yes	121	71.2
	No	49	28.8

*Those who used formal healthcare facilities were able to answer the response of the question

### Descriptive results of client, institutional and system-level variables

The results presented in Table 2 showed that 77.1% of the participants used public healthcare facilities, 54.7% made personal decisions on when and where to use healthcare, 75.3% sourced healthcare information from informal social networks, 54.1% travelled 3 or more km to access healthcare, 82.9% used car/vehicle to access healthcare facility, 61.2% spent between 20 and 40 min before they see a healthcare provider, 52.9% spent between GH¢ 50–100 anytime they utilised healthcare services, 45.3% relied on personal source of funds for healthcare, 88.8% perceived healthcare treatment as good, 90.6% ranked the quality of the healthcare as good, 91.8% rated the attitude of the healthcare provider as good, 58.2% faced communication problems and 28.8% were unsatisfied with healthcare utilisation (see Table 2).

### Client,  Institutional and System-level factors associated with formal healthcare utilisation

In Model 1 which comprised client-level variables, the results indicated that participants who relied on social protection scheme (NHIS/LEAP) as a source of funds for healthcare were 6.612 times significantly more likely to utilise formal healthcare compared with those who used personal income to cater for their healthcare expenses (AOR: 6.612; CI: 1.239–35.291). The results showed that participants who accessed formal healthcare by vehicle were 0.167 times significantly less likely to utilise formal healthcare services compared with those who accessed formal healthcare by walking (AOR:0.167; CI: 0.038–0.729). The study revealed that participants who sourced healthcare information from informal social networks were 0.206 times significantly less likely to use formal healthcare services compared with those who sourced healthcare information from a healthcare provider (AOR: 0.206; CI: 0.061–0.698). We found that participants whose family/caregivers decided on when/where to use formal healthcare were 5.841 times significantly more likely to utilise formal healthcare services compared with their counterparts (AOR: 5.841; CI: 2.080-16.404).

In Model 2 which included institutional variables plus all variables in Model 1, we revealed that participants who relied on the social protection scheme (NHIS/LEAP) to cater for healthcare expenses were 16.385 times significantly more likely to utilise formal healthcare services compared with their counterparts (AOR: 16.385, CI: 1.691–158.780). The study showed that participants who sourced healthcare information from informal social networks were 0.159 times significantly less likely to utilise formal healthcare services compared with those who sourced health information from personal sources (AOR: 0.159; CI: 0.159, 0.035–0.733). The results indicated that participants who used a vehicle to access formal healthcare had lower odds of utilising formal healthcare services (AOR: 0.101, CI: 0.015–0.658). The study revealed that participants whose family or caregivers decided on when and where to use formal healthcare services were 7.177 times significantly more likely to use formal healthcare services compared with those who decided for themselves about when and where to use formal healthcare services (AOR: 7.177; CI: 1.899–27.125). The study found that participants who perceived attitude of healthcare providers as poor were 0.575 significantly less likely to use formal healthcare services compared with those who rated the attitude of healthcare providers as good (AOR: 0.575; CI: 0.253–0.890). 

Model 3 (the final model) consisted of system variables plus all variables in Model 2. The study revealed that participants who relied on social/health protection scheme (NHIS/LEAP) to cater for their healthcare expenses were 11.934 times significantly more likely to utilise formal healthcare services compared with their counterparts (AOR: 11.934, CI: 1.151-123.777). We found that participants whose family or caregivers decided on when/where to use formal healthcare services were 12.409 times significantly more likely to use formal healthcare services compared with those who decided for themselves about when/where to use formal healthcare services (AOR:12.409; CI: 2.198–70.076). It is interesting to note that communication problem with healthcare providers was not significantly associated with formal healthcare utilisation in Model 2. However, the introduction of system variables in Model 3 revealed that participants who did not encounter communication problem with a healthcare provider were 1.358 times significantly more likely to utilise formal healthcare services compared with their counterparts (AOR: 1.358; CI: 1.074–3.737). The study found that participants who perceived attitude of healthcare providers as poor were 0.889 times significantly less likely to use formal healthcare services compared with those who rated attitude of healthcare providers as good (AOR: 0.889; CI: 0.24–0.931). We revealed that participants who spent 20–40 min at the healthcare facility were 0.070 times significantly less likely to utilise formal healthcare services compared with those who spent less than 20 minutes at the healthcare facility (AOR: 0.070; CI: 0.006–0.195) (see Table 3). The introduction of system variables such as waiting time at the health facility in Model 3 rendered the significant association between the mode of accessing healthcare and formal healthcare utilisation insignificant.

**Table 3:** System, Institutional and Client-level factors associated with formal healthcare utilisation among older adults with low income under a social protection scheme in Ghana, 2018

Variables	Model 1		Model 2		Model 3 (Full Model)	
	AOR	95%CI	AOR	95%CI	AOR	95%CI
**CLIENT PREFERENCE**						
**Source of funds for healthcare**						
Personal (ref)	1.00		1.00		1.00	
Informal social networks	***6.043***	1.303–28.024*	**6.348**	1.012–39.819*	2.743	0.368-20.433
Social/health protection scheme (NHIS/LEAP)	***6.612***	1.239–35.291*	**16.385**	1.691–158.780*	***11.934***	1.151-123.777*
**Mode of Accessing healthcare**						
Walking (ref)	1.00		1.00		1.00	
Tricycle	1.100	0.243–4.993	0.972	0.156-6.045	5.300	0.477-58.881
Vehicle	**0.167**	0.038–0.729*	**0.101**	0.015–0.658*	0.276	0.032–0.479
**Sources of healthcare information**						
Healthcare provider (ref)	1.00		1.00		1.00	
Informal social networks	***0.206***	0.061–0.698*	***0.159***	0.035-0.733*	0.194	0.037–1.016
Media	0.185	0.028–1.227	0.454	0.045–4.540	0.259	0.022–3.018
**Deciding on when/where to use healthcare**						
Personal (ref)	1.00		1.00		1.00	
Family/caregiver	***5.841***	2.080-16.404*	***7.177***	1.899–27.125*	***12.409***	2.198–70.076*
**Distance travelled to access healthcare**						
Less than 3 km (ref)	1.00		1.00		1.00	
3 or more km	2.211	0.796–6.138	2.462	0.667-9.083	1.737	0.426–7.080
**Type of healthcare provider consulted**						
Public health facility (ref)	1.00		1.00		1.00	
Private health facility	0.887	0.302–2.603	0.832	0.238-2.915	5.342	0.747–38.212
**INSTITUTIONAL**						
**Communication problems with healthcare provider**						
Yes (ref)			1.00		1.00	
No			1.695	1.196–4.460	***1.358***	1.074–3.737*
**Level of Healthcare Treatment**						
Good (ref)			1.00		1.00	
Poor			0.754	0.012–0.9154	0.122	0.001–0.437
**Quality of Healthcare Services**						
Good (ref)			1.00		1.00	
Poor			0.316	0.102–0.681	0.444	0.127–0.775
**Attitude of healthcare providers**						
Good (ref)			1.00		1.00	
Poor			**0.575**	0.253–0.890*	***0.889***	0.24–0.931*
**Satisfaction with services provided by providers**						
Good (ref)			1.00		1.00	
Poor			0.242	0.023–0.521	0.625	0.062-6.351
**SYSTEM**						
**Waiting time at healthcare facility**						
Less than 20 min (ref)			1.00		1.00	
20–40 min					***0.070***	0.006–0.195*
More than 40 min					0.198	0.016–0.495
**Healthcare Cost**						
Less than GH ¢50 (ref)			1.00		1.00	
GH¢ 50- GH¢100					0.183	0.016–2.031
More than GH¢100					0.865	0.089–8.388
**Model Fitting information**						
Percentage with correct classification	89.0		91.0		93.0	
**Omnibus Tests of Model Coefficients (sig.)**	59.360(0.000)		87.549 ((0.000)		98.550 (0.000)	
**Hosmer and Lemeshow** Chi-square **Test (sig.)**	4.352 (0.738)		4.247 (0.834)		1.943 (0.983)	
-2 Log likelihood	109.723		81.535		70.534	
Nagelkerke R Square	0.450		0.621		0.682	

Italic values indicate significance of *p*-value (*p* < 0.05); CI, confidence interval; AOR, adjusted odd ratio; * *p* < 0.05

## Discussion and implications

In recent decades, utilisation of formal healthcare by older adults, especially those with low income in developing countries has become a topical issue in international discussions. This cross-sectional study contributes to the debate by providing evidence of client, institutional and system-level factors associated with formal healthcare utilisation among older adults with low income under a social protection scheme in Ghana. In the final model, the study found that client-level factors(such as health insurance/social scheme subscription, autonomy on healthcare utilisation decision), institutional (such as communication problem with healthcare providers and attitudes of healthcare providers) and system-level factors (such as length of stay at the health facility), were associated with the use of formal healthcare among the participants. Our finding is relatively similar to evidence from previous studies [35–37]. For instance, in Ghana, van der Wielen et al. [35] reported that enrollment in NHIS among older adults increased the utilisation of inpatient and outpatient care by 6% and 9%, respectively, compared to non-members. In a study by Singh et al. [36] in India, older adults explained that long waiting times acted as a barrier to the utilisation of formal healthcare. Agyemang-Duah et al. [37] indicated that communication with health providers was associated with the use of formal healthcare among older adults.

On the client level, having health insurance increased the odds of utilising formal healthcare services compared with the lack of health insurance. Previous studies have established a positive relationship between health insurance and healthcare utilisation [38–41]. This indicates that older adults’ decision to use formal healthcare is to some extent influenced by access to health insurance considering the evidence that older adults with valid health insurance have a higher tendency to use formal healthcare compared with non-enrollees. This finding can be plausibly explained by several factors. For instance, health insurance to some extent replaces out-of-pocket payment for formal healthcare services, which enhances access to health services at a reduced or no cost. Because our study participants were older adults with low income, the removal or reduction of financial barriers to healthcare allows them to use services since the cost burden is either removed or diminished. This explanation is supported by previous assertions and observations by WHO [42] and Van der Wielen et al. [35] that social protection schemes such as health insurance promote healthcare utilisation among groups with low income including older adults. More importantly, our finding indicates that health insurance is a dominant client-level factor influencing the use of formal healthcare among older adults with low income. This is because the introduction of institutional and system-level factors such as waiting time, quality of care, satisfaction and communication problems did not significantly reduce the strength of association between health insurance enrolment and healthcare utilisation. This somewhat supports the proposition that financial constraints could be, perhaps, the major reason, for unmet needs for, and delays in, seeking healthcare and reduced well-being in later life [11, 43–46]. Our finding, therefore, implies that policies and measures to improve the implementation and sustainability of Ghana’s health insurance scheme are imperative. This is in line with Gyasi et al. [47] observation that when well-resourced and systematically sustained, Ghana’s health insurance scheme could eventually lead to improvements in the health status of older persons due to the improvement in healthcare use, insofar as these two variables are positively associated.

The study revealed that autonomy concerning decisions as to when and where to seek healthcare as a client-level factor was also associated with healthcare utilisation among older adults with low income. Interestingly, those whose family/caregivers decided on where and when to use formal healthcare had a greater tendency to use formal healthcare compared to those who could decide for themselves. This finding is interesting and appears somewhat difficult to explain. However, it would relate well with the sociocultural practices in Ghana that promote collective decision making about health [48; 49]. Also, it is possible that most of the participants might have developed dementia and other forms of Alzheimer’s disease which keep their thought from the use of formal healthcare services [50], especially when they do not have any family/caregiver to determine when and where to utilise healthcare services. Again, it is possible that most of the participants have developed strong taste for traditional medicine as remedies for their health problems, hence autonomy concerning decisions as to when and where to seek help veer towards the use of tradional medicine. We however acknowledge that the above reasons may not be sufficient as there is the likelihood of other underlying factors to predict the lower odds of formal healthcare utilisation among the participants who have autonomy in relation to formal healthcare utilisation. However, because of the quantitative nature of this study, we were not able to look into this issue which will call for a further qualitative study.

Crucially, perceived communication barrier was an important institutional-level factor that influence formal healthcare utilisation among the participants. Older adults who did not encounter communication problems with healthcare providers had higher odds of utilising healthcare services compared to those who experienced communication problems with providers. Previous studies have highlighted the important roles of effective communication in health provision [51–55]. Evidence shows that effective communication among patients and providers is essential in all healthcare settings and all aspects of healthcare, from prevention and health maintenance to illness diagnosis, treatment, and recovery [56–58]. Research also shows that effective communication, grounded by core values, improves health outcomes, quality of care, and patient and clinician satisfaction [59]. Likewise, the link between poor communication and poor health outcomes has been extensively established [60]. Poor communication in healthcare leads to delayed treatment [60]. Without effective communication, messages and signals will be misinterpreted and misunderstood which can lead to unmet needs of patients [61]. This suggests that perceived communication problem with providers serves as a barrier to the use of formal healthcare services among older adults [62]. To facilitate greater use of formal healthcare by older adults, healthcare organisations and providers should identify ways to promote effective cross-cultural communication in healthcare settings [62].

Our study also found that another institutional-level factor associated with older adults’ formal healthcare use was perceived poor attitude of healthcare providers. Older adults who perceived the attitude of healthcare providers as poor had decreased odds of utilising formal healthcare compared to those who rated the attitude of healthcare providers as good. Healthcare providers’ attitude toward older adults has been previously mentioned as an important determinant of formal healthcare use [43, 62, 63]. Evidence suggests that the physiological and psychological conditions of older adults require that healthcare providers treat them with maximum care and respect [63]. However, many older adults mostly do not get the expected care and respect from providers, and this influences their decision to utilise formal healthcare [62]. This may explain why in our study older adults who perceived providers’ attitudes to be poor were less likely to utilise formal healthcare services. Personal experiences and previous encounters among our participants may also explain this finding. For instance, in Namibia, Aboderin and Beard [64] reported that older patients did not use commercial providers because of the unavailability, perceived poor quality, or age insensitivity of services in government facilities. Also, in a qualitative study by Agyemang-Duah et al. [62] in Ghana, older adults explained that non-respectful attitude, unapproachable interaction style and failure of formal healthcare providers to accord them the needed respect and care influence their decision to stay away from formal healthcare utilisation. Our finding, therefore, indicates that a change in providers’ attitudes may improve formal healthcare utilisation among older adults with low income in Ghana.

Again, this study found that system level factors such as waiting time was associated with formal healthcare utilisation among older adults with low income. Older adults who spent between 20 and 40 min at the healthcare facility had decreased odds of utilising formal healthcare compared with those who spent less than 20 min. Waiting time has been previously identified as a system-level issue that significantly influences healthcare utilisation [64, 65]. Our finding suggests that long waiting times at a health facility may influence older adults’ decision to utilise formal healthcare. To address long waiting times, healthcare providers should employ adequate and competent staff to care for patients [43, 66]. On the client level, older adults should visit health facilities near them, on days other than Mondays and in the afternoons to avoid long waits at health facilities [67].

To the best of our knowledge, this is one of the first studies that explore this important issue, especially for older adults with low income enrolled in a social protection scheme in Ghana. Despite the contributions of this study, some limitations are notable. Our findings may be exposed to a potential selective survival bias which may partly be attributed to the selection procedure of the sample and criteria for defining, conceptualising, or categorising our variables and key concepts in this study. Also, given the cross-sectional nature of the study, the direction of the causal relationship between healthcare use and factors influencing healthcare utilisation among older adults with low income cannot be determined. The results only show a significant association between these variables. Future studies should investigate the causality of the associations and the underlying mechanisms in more detail.

### Implications for policy, practice and Future Research

This study provides several contributions to policy discussions and research literature concerning system, institutional and client-level factors associated with formal healthcare utilisation among older adults with low income in a LMIC country. In addition, the study contributes to the achievement of Ghana’s ageing policy goal of social, economic, and cultural reintegration of older people into mainstream society. Older people are entitled to access to healthcare services to maintain optimum levels of physical, mental and emotional well-being. The findings of this study, therefore, provide input for Ghana’s ageing policy to promote lifelong health. Our finding that multifaceted and multi-level factors that are systematic, institutional, and individualistic in nature influence formal health care use of older adults specifically those within the poor category require either a change in policy and practice or the development and implementation of policies and interventions to address these issues. Policy(ies) and programmes concerning older adults in Ghana should acknowledge that factors affecting older adults’ formal healthcare use are both systemic and institutional and not only individual-level issues. This acknowledgement will promote holistic, comprehensive, and ageing-sensitivity programmes and policies. Moreover, the findings of our study imply that more research is needed in this important research to adequately offer evidence that influences policy and practice.

## Conclusion

This study examined the effect of system, institutional and client-level factors on formal healthcare utilisation among older adults with low income under a social protection scheme in Ghana. We argue that specific system, institutional and client-level factors contribute to formal healthcare utilisation among older adults with low income. The take-home messages based on our findings are highlighted: (1) older adults with low income who relied on a social protection scheme specifically the LEAP programme and or health insurance subscription to cater for healthcare expenditure had higher odds of using formal healthcare (2) participants who did not encounter communication problem with healthcare providers were significantly more likely to utilise formal healthcare services (3) participants who did not decide on when/where to use formal healthcare were more likely to utilise formal healthcare services (4) those who perceived the attitude of healthcare providers as poor were significantly less likely to utilise formal healthcare (5) those who spent 20–40 min at the healthcare facility were significantly less likely to utilise formal healthcare services compared with their counterparts. We recommend that reducing waiting time at the healthcare facility, improving the social protection scheme, improving patient-doctor communication at the health facilities and attitudinal change programmes (such as orientations and supportive supervision) of healthcare providers may help to increase formal healthcare utilisation among older adults with low income in Ghana.

## Data Availability

The datasets generated and/or analysed during the current study are not publicly available because other manuscripts are yet to be developed from the datasets but are available from the corresponding author upon a reasonable request.
