# Correction: System, institutional, and client-level factors associated with formal healthcare utilisation among older adults with low income under a social protection scheme in Ghana

**DOI:** 10.1186/s13690-023-01113-3

**Published:** 2023-05-26

**Authors:** Williams Agyemang-Duah, Dennis Asante, Joseph Oduro Appiah, Anthony Kwame Morgan, Isaac Verberk Mensah, Prince Peprah, Anthony Acquah Mensah

**Affiliations:** 1 Department of Geography and Planning, Queen’s University, Kingston, Canada; 2 College of Medicine & Public Health, Rural and Remote Health, Flinders University, Adelaide, South Australia Australia; 3 Department of Geography, Environment & Spatial Analysis, Cal Poly Humboldt, Arcata, CA USA; 4 Department of Planning, Kwame Nkrumah University of Science and Technology, Kumasi, Ghana; 5 Department of Social Sciences, St. Ambrose College of Education, Wamfie, Bono Region Ghana; 6 Social Policy Research Centre, Centre for Primary Health Care and Equity, University of New South Wales, Sydney, Australia

**Correction:** ***Arch Public Health *81,** **68 (2023)**

10.1186/s13690-023-01063-w

Following publication of the original article [1], the authors reported that three paragraphs should be updated to correct the grammatical mistakes.

In paragraph 2 in the Discussion section, the original texts were:

This explanation is supported by previous assertions and observations by WHO [42] and Van der Wielen et al. [35] that social protection schemes such as health insurance promote healthcare utilisation in groups and communities with low income for older adults and other vulnerable groups such as women and children.

The corrected texts should read:

This explanation is supported by previous assertions and observations by WHO [42] and Van der Wielen et al. [35] that social protection schemes such as health insurance promote healthcare utilisation among groups with low income including older adults.

In paragraph 4 in the Discussion section, the original texts were:

Older adults who did not encounter communication problems with healthcare providers had higher odds of utilising healthcare services compared to those who experienced communication providers with providers.

The corrected texts should read:

Older adults who did not encounter communication problems with healthcare providers had higher odds of utilising healthcare services compared to those who experienced communication problems with providers.

In paragraph 1 in the Conclusion section, the original texts were:

We argue that specific system, institutional client-level factors contribute to formal healthcare utilisation among older adults with low income.

The corrected texts should read:

We argue that specific system, institutional and client-level factors contribute to formal healthcare utilisation among older adults with low income.

The original article [1] has been updated.
